# Microfluidic System for In Vivo-Like Drug Permeation Studies with Dynamic Dilution Profiles

**DOI:** 10.3390/bioengineering8050058

**Published:** 2021-05-05

**Authors:** Thomas Lorenz, Mona Kirschke, Verena Ledwig, Stephan Reichl, Andreas Dietzel

**Affiliations:** 1 Center of Pharmaceutical Engineering, Technische Universität Braunschweig, 38106 Braunschweig, Germany; t.lorenz@tu-braunschweig.de (T.L.); mona.kirschke@tu-braunschweig.de (M.K.); v.ledwig@tu-braunschweig.de (V.L.); s.reichl@tu-braunschweig.de (S.R.); 2 Institute of Microtechnology, Technische Universität Braunschweig, 38106 Braunschweig, Germany; 3 Institute of Pharmaceutical Technology and Biopharmaceutics, Technische Universität Braunschweig, 38106 Braunschweig, Germany

**Keywords:** drug testing, in vitro, sodium fluorescein, MDCK, microfluidic test system, organ-on-chip, permeation, transepithelial electrical resistance, TEER

## Abstract

Automated biomimetic systems for the preclinical testing of drugs are of great interest. Here, an in vitro testing platform for in vivo adapted drug absorption studies is presented. It has been designed with a focus on easy handling and the usability of established cell cultivation techniques in standard well plate inserts. The platform consists of a microfluidic device, which accommodates a well plate insert with pre-cultivated cells, and provides a fluid flow with dynamic drug dilution profiles. A low-cost single-board computer with a touchscreen was used as a control unit. This provides a graphical user interface, controls the syringe pump flow rates, and records the transepithelial electrical resistance. It thereby enables automated parallel testing in multiple devices at the same time. To demonstrate functionality, an MDCK cell layer was used as a model for an epithelial barrier for drug permeation testing. This confirms the possibility of performing absorption studies on barrier tissues under conditions close to those in vivo. Therefore, a further reduction in animal experiments can be expected.

## 1. Introduction

The development of new drugs is a complex and scientifically demanding task that is also time consuming and costly [1,2]. Only a few of the most promising new drug candidates make it through the clinical test phase successfully and are introduced to the market as approved drugs [3]. To minimize the failure of potential candidates in the most expensive clinical tests, the use of a valid and significant test system in preclinical tests is of particular importance. For testing new drugs in preclinical studies, animal models are traditionally used. Since animal models are expensive, difficult to transfer to humans, and—above all—ethically questionable, they are increasingly being replaced by in vitro cell culture models [4]. However, most of the well-established in vitro systems are static and therefore do not have the ability to mimic human in vivo environments, which is detrimental for a reliable prediction of substance behavior in the subsequent clinical studies.

In recent years, microfluidic organ-on-chip systems have been studied, and they promise to create organotypic environments [5]. The resulting challenge is to transfer established, static, in vitro tissue models to novel microfluidic systems that enable dynamic processes. However, automation and parallelization are two major challenges for the acceptance of current cell-based microfluidic systems [6].

A testing strategy has to be developed that needs fewer or even no animal models, resulting in more reliable preclinical testing and at best increasing the predictability of promising drug candidates. In particular, endothelial and epithelial absorption barriers would benefit from a microfluidic test option. For example, in an in vitro model of the blood–brain barrier, microfluidic conditions affect endothelial cells, resulting in a higher barrier through the intensified formation of tight junctions [7]. Tear flow can be simulated in a microfluidic model of the cornea, which is of particular interest for absorption studies to simulate in vivo conditions [8]. Other in vitro models, such as those of the intestine, nasal mucosa, skin, or lung also offer improved testing conditions through microfluidics [4,9,10,11]. The more organotypic test conditions also lead to significantly different responses to pharmaceutical agents compared to static test conditions [5]. Further, various tissues have been shown to remain viable under microfluidic conditions for a longer period of time [12]. To mimic in vivo conditions even more closely, the flow and the changing concentration profile due to absorption and dilution processes need to be taken into account. Thus, a system is needed that allows the transfer from a static to a dynamic dilution in order to create conditions that are close to in vivo conditions and to obtain more meaningful test results.

A major challenge in the use of microfluidic organ-on-chip systems is the cultivation of cells inside the devices. The microfluidic test system already described in earlier works addresses this problem by combining well-established static models and static cultivation with microfluidic tests under organotypic conditions [8,13,14]. It was initially developed to simulate the dilution of drugs applied to the eye in an in vitro model and analyze the drug permeation [13]. Further, it has been used to study dynamic cell culture conditions of an in vitro blood–brain barrier model and its barrier properties [14]. These dynamic models aim to provide an in vivo-like fluid flow across the cellular surface.

In these studies, the concentration profile was mimicked by first filling the donor flow channel of the fluidic device with the active ingredient solution diluted with a buffer solution, which was delivered from one syringe pump. The volume ratios of the donor and acceptor compartments were designed in accordance with the fluid volumes in the in vivo situation, and the flow rates were also selected to represent that ratio. To monitor the cell barrier, stainless steel electrodes for transepithelial electrical resistance (TEER) measurements were installed in the upper and lower levels of the system.

Nevertheless, several unsolved problems remained. The actual time course of the concentration in the donor compartment could not be determined, as it depended not only on the volumes and flow rates but also on a lack of mixing in the laminar regime with interfacial and surface tensions depending on the different materials used. Additionally, the initial filling of active ingredient solution had to be performed manually, which prevented parallelization of experiments. To make a decisive step in replacing established tissue cultures in preclinical testing and to better predict clinical outcomes, organ-on-chip systems must include loading with programmable pre-mixing with the desired time course of the active substance concentration. Therefore, in our study we aimed to develop an advanced system that allows automated in vivo adapted permeation of drug candidates while providing the ability to continuously measure and record TEER as a quality criterion for cultured barrier tissues. Only with such automation and continuous recording of quality parameters can the necessary reliability be generated that is required in the preclinical testing of pharmaceutical products, as well as for the acceptance of such an animal test replacement method by the regulatory authorities.

## 2. Materials and Methods

### 2.1. Materials and Setup

The long-term goal of this project is to develop a system that is able to replace animal testing during drug development, especially in pre-clinical permeation studies. The microfluidic system not only needs to generate in vivo-like conditions but also has to provide a high degree of automation that is easy to handle, as well as being cost-efficient.

#### 2.1.1. Microfluidic Device

**Figure 1:**
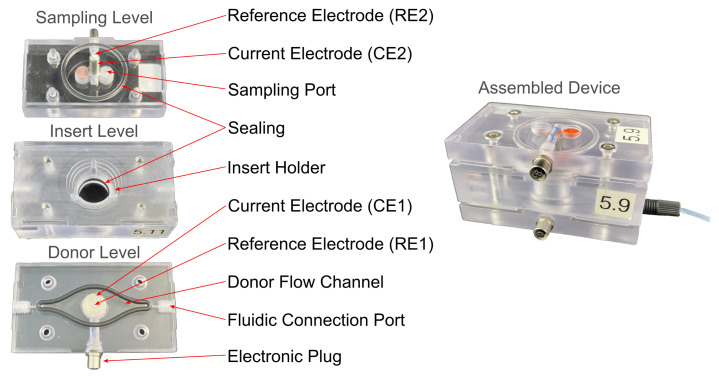
Photo of the fluidic device levels and the assembled device. Outer dimensions: w=7.3 cm, l=4.0 cm, h=3.8 cm.

Like the device in prior work [8], the new system (Figure 1) is manufactured from polycarbonate (PC) and consists of three levels. The bottom level contains a flow channel, which is connected to the syringe pumps on the inlet and to a waste container on the outlet. This level has been remodeled by integrating the fluid connection ports, which were separate components with additional O-ring seals mounted by screws in the earlier design. Further, a standard O-ring has been introduced as a seal around the flow channel for leak tightness. The middle insert carrier level was improved by relocating the position of the O-ring, which guarantees that there is no fluid transport around the membrane of the insert, and by adjusting its dimensions. The latter was necessary to account for the fabrication tolerances of the membrane inserts. The top level closes the device to prevent evaporation and allows sampling with a syringe and a needle through a septum.

#### 2.1.2. Transepithelial Electrical Resistance Measurement

Automated and integrated TEER measuring electronics have been developed to allow for continuously repeated recordings of TEER values for up to four devices in parallel. A STEMlab 125-14 from Red Pitaya is utilized as a signal generator and an oscilloscope in combination with an electronic circuit for a potentiostat, which was adapted from Kalinowski et al. [15] to follow the low-cost concept of our setup. The STEMlab used is a field-programmable gate array (FPGA), which can be used as a multifunctional laboratory instrument [16]. It can either be operated via a browser-based GUI or by SCPI commands. The latter was used to remotely control the STEMlab from our master unit.

**Figure 2:**
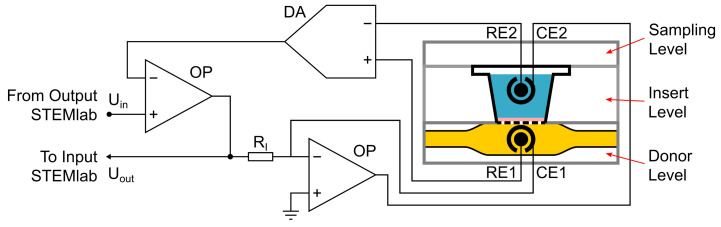
Electronic circuit of the potentiostat used for automated TEER measurements in connection to the microfluidic device. OP = operational amplifier; DA = differential amplifier; R_I_ = shunt resistor; CE = current electrode; RE = reference electrode.

The analog output of the STEMlab is used as an input signal for the potentiostat circuit (see Figure 2). It is also connected to one of the analog inputs of the STEMlab. The amplitude and frequency of a sinusoidal voltage are set in the STEMlab via an SCPI command from the control unit. A frequency of f=13 Hz and a peak-to-peak voltage of $V_{pp}=130\;\mathrm{mV}$ are typically selected. These values are also used in standard TEER measuring equipment such as the EVOM2 (World Precision Instruments) and the cellZscope (nanoAnalytics). As shown in Figure 2, the input is connected via the operational amplifiers (OPs) to the current electrodes (CEs) on both sides of the membrane with a cell layer. Two reference electrodes (REs) are also located at both sides of the membranes, as described earlier [8]. These REs are connected to a differential amplifier (DA) consisting of three OPs and fed back to the input signal. This prevents any drift in potential, which is a typical problem in electro-chemical measurements. The output signal is collected in front of a shunt resistor and is connected to an analog input of the STEMlab.

During a measurement, the output voltage of the potentiostat is recorded by the STEMlab for a defined duration, and the measured values are sent to the control unit. Fourier transformation reveals amplitudes and phases in dependency of frequency. These values are then used for the calculation of the TEER value.

#### 2.1.3. Automated Test Setup

**Figure 3:**
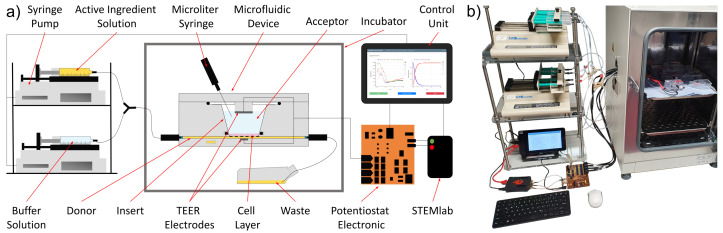
Automated test setup. (**a**) Schematic of the experimental setup and connections. (**b**) Photo of the experimental setup.

Two syringe pumps KDS 220 (KD Scientific, Holliston, MA, USA) that allow for the parallel use of up to 10 syringes each (depending on the syringe volume and size) were used for the fluid supply of the buffer solution and the drug/test solution. One pump was loaded by a syringe filled with the active-ingredient solution, and the other pump was loaded with a buffer solution. The fluids inside the syringes were heated to 37 ${}^{\circ }C$ prior to filling. Both syringes were connected to a Y-junction, and the combined fluid flow was connected to the microfluidic device with a 1 m tube to allow for the proper mixing of the buffer and drug by diffusion before reaching the cells inside the device. The device was placed in a temperature-controlled incubator at 37 ${}^{\circ }C$. The outlet of the device was connected to a drain bottle using a short tube with a greater diameter of 1 mm to avoid the build-up of back pressure. The complete setup is shown in Figure 3.

**Figure 4:**
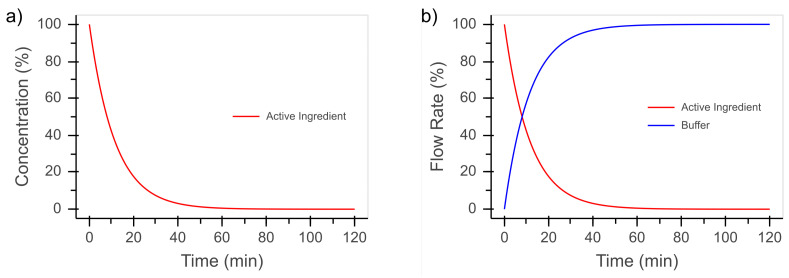
(**a**) Concentration profile according to Equation (1). (**b**) Deduced flow rates to mimic the concentration profile with two pumps, of which one contains the active-ingredient solution and the other contains a buffer solution.

As an example for a complex time course of concentration, the dynamics of the pre-corneal residence time was chosen, which is significantly influenced by tear flow. Therefore, a dynamic dilution of $0.083\min^{-1}$ was chosen for our study, as this corresponds to the basal tear flow rate [17]. The concentration on the apical side of the eye can be described by

(1) $$c\left(t\right)=c_{0}\;\cdot \;{\left(1-p\right)}^{t}$$

with p=0.083, as shown in Figure 4a. To prevent any influence of the flow rate and the resulting pressure change in the fluidic device on the permeation process, a constant flow rate is desired. The flow rate of the active ingredient pump starts with 100% of the total flow rate and decreases by $8.3\%\min^{-1}$. The buffer pump starts at 0% of the total flow rate and increases by $8.3\%\min^{-1}$. These flow rates are shown in Figure 4b.

Most conventional laboratory pumps do not allow one to directly program a complex flow profile, or they only allow for the programming of linear flow rate changes. However, the syringe pumps we used (as per most other laboratory pumps) can be controlled via RS232. This enables the pumps to perform any flow profile by transmitting the desired flow rate to the pumps at a regular interval. An additional control unit and software enabling serial communication are required for this purpose. A Raspberry Pi 4 Model B (Raspberry Pi Foundation, Cambridge, UK) with 2 GB of RAM and a USB to RS232 converter was used as a master unit. It was equipped with a 7 inch touchscreen (Raspberry Pi Foundation, Cambridge, UK) to allow for usage even without additional input devices such as a mouse or keyboard. The RasClock by Afterthought (Bristol, UK) module was added to the GPIO pins of the Raspberry Pi to ensure accurate time data even without an internet connection. All the components were integrated in a housing (SmartiPi Touch 2 by Smarticase, Philadelphia, PA, USA) for proper handling and safety reasons. Additionally, the STEMlab was connected to the Raspberry Pi via Ethernet and to the potentiostat PCB via its coaxial analog in- and outputs.

#### 2.1.4. Control Software and Graphical User Interface

Software was written in Python to allow for the delivery of an exponentially decreasing concentration profile to the fluidic device and to automate the experiments as much as possible. The software was also aimed at the easy handling of the experimental setup by providing an intuitive graphical user interface (GUI) to the user. Further, automated recording of the experimental data for quality assurance was introduced to make the fluidic system suitable for the drug approval process. The software uses the web framework Flask [18] for the GUI. A browser-based software design was chosen to easily allow for the remote control of the experimental setup and enable its usage on almost any device. The software is structured in four parts, which resemble its main tasks: the user interface, the control of and communication with the pumps, and the measurement of the TEER values. The fourth task is the recording and documentation of all setup settings and measurement data and extends over all of the aforementioned parts.

**Figure 5:**
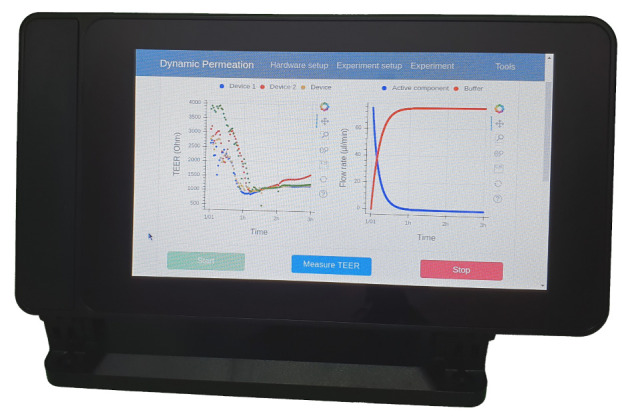
Measurement software with the graphical user interface (GUI) running on the RaspberryPi control unit.

When starting the software, an input window opens, where all important experimental parameters need to be entered. This includes details on the flow and concentration profile, the pumps and syringes, and the TEER measurement. These parameters are stored in an SQL database and can be loaded for another experiment later on. The chosen values are also written to the header of the record file in combination with the current time and date. The record file is stored in CSV format (Comma Separated Values) to allow usage of the data in almost every type of evaluation software. After this setup procedure is finished, the experiment window opens (Figure 5) and shows the measured TEER values over the experiment time and the flow rates of both pumps. Before starting the dynamic experiment, the TEER values of the cells in the fluidic devices can be measured. When starting the experiment, the pumps deliver the flow rates according to the set parameters, and the TEER values are measured in the set intervals until the experiment is stopped manually or the set duration is over. Pump rates and TEER values are written to the record file.

#### 2.1.5. Chemicals

Phosphate-buffered saline (PBS), fetal bovine serum (FBS), and Earle’s Minimum Essential Medium with $2.2\;g\;L^{-1}$ NaHCO_3_ and 2 mM l-glutamine were received from Biochrom (Berlin, Germany). Sodium fluorescein and antibiotic solution with 10,000 $U\;{\mathrm{mL}}^{-1}$ penicillin, 10 $\mathrm{mg}\;{\mathrm{mL}}^{-1}$ streptomycin sulfate, and 25 $\mu g\;{\mathrm{mL}}^{-1}$ amphotericin B were purchased from Sigma-Aldrich (Taufenkirchen, Germany). EDTA disodium salt solution 2% was purchased from MP Biomedicals (Santa Ana, CA, USA). Trypsin-EDTA (0.50.2 g L−1) was obtained from Thermo Fisher Scientific (Waltham, MA, USA). The Krebs–Ringer buffer (KRB) contained 6.8 g of sodium chloride, 3.575 g of HEPES, 2.1 g of sodium bicarbonate, 1.1 g of d-glucose monohydrate, 0.4 g of potassium chloride, 0.26 g of calcium chloride dihydrate, 0.2 g of magnesium sulfate heptahydrate, and 0.14 g of sodium dihydrogen phosphate monohydrate per 1000 mL of double-distilled water. The pH was adjusted to 7.4.

#### 2.1.6. Materials

Cell culture flasks were obtained from Sarstedt (Nümbrecht, Germany). Twelve-well plates and Transwell inserts (art. no. 3460) were bought from Corning Costar (Kennebunk, ME, USA). Multi-syringe infusion pumps *KDS 220P* by KD Scientific (Holliston, MA, USA) were used in the experimental setup in combination with single-use Inkjet LL syringes with a volume of 5 mL and 20 mL by B. Braun (Melsungen, Germany). PTFE-tubing with an outer diameter of 116 inch and inner diameters of 0.25 mm and 1 mm were purchased from Techlab (Braunschweig, Germany). Fittings, ferrules, and Y-junctions by IDEX Health & Science (Oak Harbor, WA, USA) were used. EVOM2 in combination with an EndOhm-12G chamber electrode by World Precision Instruments (Sarasota, FL, USA) was used for comparative TEER measurements. An incubator and 96-well cell culture test plates (black) were received from Thermo Fisher Scientific (Witham, UK). A fluorescence microplate reader GeniOS from Tecan (Männedorf, Switzerland) was used for fluorescence measurements.

### 2.2. Methods

#### 2.2.1. Cultivation of Madin Darby Canine Kidney Cells

The Madin–Darby canine kidney (MDCK) cell line originated from the renal duct of an adult female cocker spaniel. MDCK type I cells at Passage 17 were received from the European Collection of Authenticated Cell Cultures (ECACC). This cell line grows fast on filters and forms tight monolayers with a polarized morphology. Cells were cultivated in Earle’s MEM containing $2.2\;g\;L^{-1}$ NaHCO_3_, 10% (*v/v*) FBS, 2 mM l-glutamine, and 1% (*v/v*) antibiotic/antifungal mixture in 25 cm${}^{2}$ cell culture flasks at 37 ${}^{\circ }C$ under a humidified atmosphere with 5% CO_2_. The growth medium was replaced three times per week. When reaching confluence, cells were counted by using a Vi-CELL XR Analyzer from Beckman Coulter (Krefeld, Germany), subcultured and seeded into the transwell inserts with a cell density of $0.4\times 10^{6}$ $\mathrm{cells}\;{\mathrm{cm}}^{-2}$. In the inserts, the cells were cultured for an additional 5 days until they formed a sufficiently high TEER for the permeation experiments. The growth medium was changed on cultivation days 3 and 4. Permeation was performed on the 5th day of cultivation.

#### 2.2.2. Dynamic Concentration Verification

In order to verify that the pumps delivered the desired volumes to obtain the specified dilution profile, the pumped volumes were collected for 60 min at different time points, and the mass of the extracted fractions was determined. In addition, it was checked whether the desired donor concentration was present at different time points in the dilution profile. The donor was collected at the outlet of the system and accumulated in 5 min fractions; $2.5\;\mathrm{mg}\;{\mathrm{mL}}^{-1}$ sodium fluorescein was used as the donor with a dilution profile of 8.3% per minute. The samples were analyzed with a fluorescence microplate reader at an excitation wavelength of 485 nm and an emission wavelength of 535 nm.

#### 2.2.3. Determination of the Cellular Barrier

To evaluate the epithelial barrier properties of MDCK cell layers, the apparent permeation coefficient (P_app_) of sodium fluorescein as a marker substance was determined in addition to the TEER measurement. For this purpose, the permeation coefficient was determined from a static experiment in a well plate as well as with an experiment in the fluidic device with a flow rate of 75 $\mu L\min^{-1}$ (details on the experimental setup are described in Section 2.2.4). Both experiments were performed without a dynamic dilution using a sodium fluorescein concentration of $2.5\;\mathrm{mg}\;{\mathrm{mL}}^{-1}$. To calculate the P_app_ [19], the linear part of the permeation profiles from 5 min to 60 min was used. The permeation coefficient was determined to prove that the barrier of the MDCK monolayer in the static experiments in the well plates and in the microfluidic device under flow are comparable.

#### 2.2.4. Permeation Studies

Before the permeation was started, the cellular layer was incubated with KRB (500 μL apical and 1500 μL basolateral) for 30 min. Afterwards, TEER was measured in the Endohm chamber with the new TEER electronics. In the case of static experiments (see Section 2.2.3), a solution of sodium fluorescein ($2.5\;\mathrm{mg}\;{\mathrm{mL}}^{-1}$) in KRB (200 μL) was used as a donor, and 900 μL of KRB served as the acceptor. For experiments under flow conditions, the respective inserts were transferred to the microfluidic device. Aliquots (90 μL) were taken from the acceptor at fixed time intervals for 180 min, and the withdrawn volume was replaced with tempered KRB (37 ${}^{\circ }C$). For the permeation study with dynamic donor dilution, the experimental parameters were chosen in the context of testing drugs for application to the eye. According to the natural turn-over rates at a basal lacrimal flow, an absolute dilution of 31 $\mu L\;\min^{-1}$ in the fluidic device was obtained at a drug dilution of $8.3\%\min^{-1}$ [17], related to the volume of the lower-level flow channel (370 μL). The total flow rate Q_total_ of the donor was set to 75 $\mu L\min^{-1}$ for all experiments with new parameters. This flow rate led to a small time delay between the activation of the syringe pumps with new parameters and the adjusted concentration reaching the cells.

## 3. Results

### 3.1. Dynamic Concentration Profile

**Figure 6:**
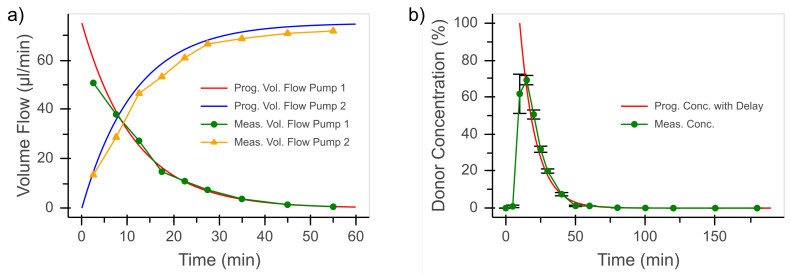
(**a**) Comparison of the programmed and experimentally determined volume flows of Pumps 1 and 2. (**b**) The programmed donor concentration profile according to Equation (1) with a time delay (10 min) compared to the measured donor concentration profile compared to the desired concentration profile (n = 2).

At a dilution profile with $8.3\%\min^{-1}$ and a total flow rate of 75 $\mu L\;\min^{-1}$, the calculated target volumes correspond to the volumes of Pumps 1 and 2 determined experimentally (Figure 6a).

Subsequently, it was verified that the desired donor concentration reaches cells in the experiment at specific time points. Since the system is flushed with KRB buffer before the start of the experiment, the expected concentration becomes effective with a delay. In Figure 6b, the concentration profile with a 10 min time delay corresponds very well with measurements. This time lag and the initial dilution of the donor concentration (due to the KRB-filled system) should not affect the significance of the results obtained.

### 3.2. Transepithelial Electrical Resistance

**Figure 7:**
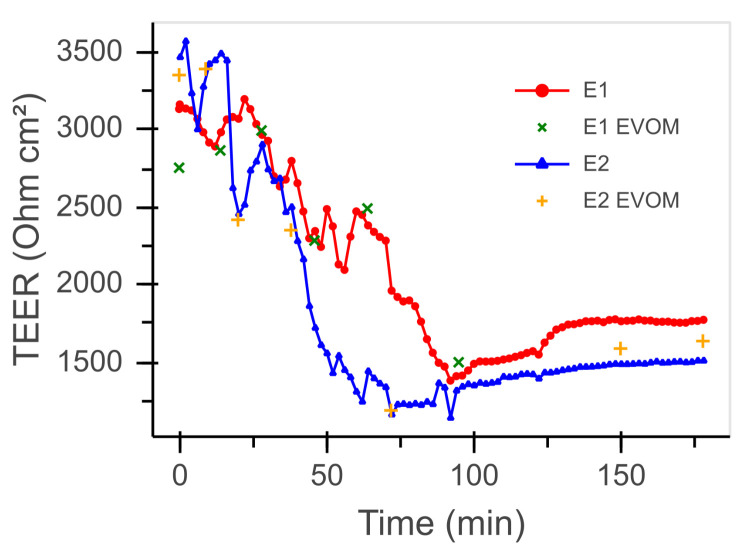
Automatically recorded TEER values for two permeation experiments (E1 and E2) in comparison to values recorded using the EVOM2.

Exemplary TEER recordings from the permeation experiments are shown in Figure 7. In intervals of 2 min, the TEER values of the different devices were recorded. Additionally, spot measurements with the EVOM2 are shown for comparison.

In both experiments E1 and E2, an initial TEER value of 3000 to 3500 Ω cm^2^ was observed at the beginning of the measurements. Upon the start of the dynamic flow conditions, the TEER value dropped steadily within the first 90 min to levels in the range of about 1500 Ω cm^2^. In the course of the following 90 min, the TEER value of the MDCK cell layer stabilized and even exhibited a slight increase to values between 1500 and 1700 Ω cm^2^. For the TEER values obtained, very good agreement was found between the data recorded by the newly developed continuous measuring system and those determined by the standard laboratory instrument EVOM2.

### 3.3. Permeation Studies

The P_app_ of sodium fluorescein was determined in addition to the TEER to evaluate the epithelial barrier properties of the MDCK monolayer. In the comparison of the permeability of the MDCK cell layer under static experimental settings in the well plates and under dynamic conditions in the fluidic device, no significant differences were found (Student’s *t*-test, *p* < 0.05) with P_app_ measured in well plates $5.422\times 10^{-8}\;\mathrm{cm}\;s^{-1}$ (± $1.316\times 10^{-8}\;\mathrm{cm}\;s^{-1}$; n = 11) and P_app_ in the fluidic device $5.261\times 10^{-8}\;\mathrm{cm}\;s^{-1}$ (± $7.160\times 10^{-9}\;\mathrm{cm}\;s^{-1}$; n = 4). It can be assumed that the permeation barrier in the newly designed test setup with the application of a flow is comparable to the barrier in the usual static test.

**Figure 8:**
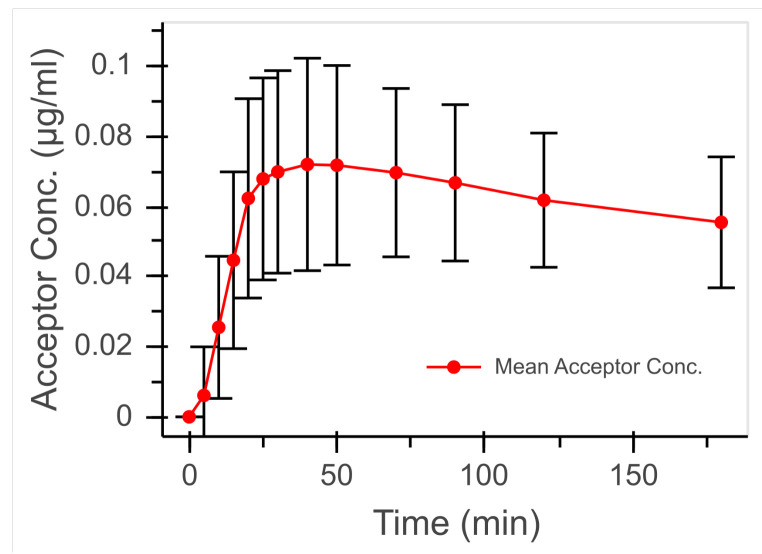
Mean acceptor concentration with standard deviation from experiments with dynamic dilution (n = 18).

Based on repeated experiments (n = 18), the course of the permeation was measured as the acceptor concentration over time (Figure 8). The comparatively high proportion of the active-ingredient solution at the beginning of the experiment led to a steep increase in the acceptor concentration. This increase flattened as the experiment progressed with a decreasing proportion of drug solution in the total flow rate before reaching a maximum ( $0.072\;\mu g\;{\mathrm{mL}}^{-1}$ at 40 min). After the maximum was reached, a continuous slow decrease in the acceptor concentration until the end of the experiment was observed.

## 4. Discussion

The introduced two-pump method is not limited to the presented exponential profile, and it allows arbitrary profiles through software settings. The overall flow rate can be selected independently to the experimental needs. In our case, it was chosen to be 75 $\mu L\;\min^{-1}$, as this was shown to be a good compromise between the reasonably short delay time of the delivered concentration and the absence of pressure built up inside the device. The automation of the fluid delivery allowed us to run up to four experiments simultaneously using multi-syringe pumps. This tackled the issue of the parallelization not yet being resolved in most current cell-based microfluidic systems [6] and will even be extended in the future. The bottleneck for further parallelization is currently the sample taking from the acceptor volume, which is still performed manually to avoid any cross-contamination due to the small sample volume. This challenge is addressed in our ongoing research.

The graphical user interface allows for the repeatable execution of the experiments. The continuous recording of the TEER might reveal especially interesting information on the condition of the cell layer. Up to now, the EVOM2 by World Precision Instruments has been accepted as the standard measuring device for cell layer TEER, but it only provides a simple on/off switch to start the measurement. EVOM3 has just been released, but it still does not allow for continuous, automated, or parallel measurements. The cellZscope by nanoAnalytics, another standard instrument for the determination of TEER, is designed for parallel and automated measurements of multiple well plate inserts only under static conditions. This device also allows for the recording of an impedance spectrum, which can provide additional information of the cell layer [20]. For integrated impedance measuring in a cells containing microfluidic systems, the use of a potentiostat/galvanostat is preferable. This is the most flexible method, as it allows for recording at a single frequency or over a complete spectrum [21]. On the downside, potentiostats/galvanostats are still very expensive, and additional electronics, software, or both, are necessary for parallel use in more than one system. In our low-cost and easy-to-use approach, the electronic circuit for the potentiostat is relatively simple in its design and can be manufactured using standard electronic components. In the future, the STEMlab 125-14 could be replaced by a microprocessor that handles the signal generation and analog signal detection, which would decrease equipment costs even more. However, advanced knowledge would be needed to flash the software to the microprocessor, which would increase the work required for this setup to be replicated and used in pre-clinical test laboratories.

In the TEER profiles shown in Figure 7, the continuously recorded TEER values are generally in good agreement with the EVOM2 measurements. The few deviations of the two different measuring methods could be due to several reasons. The EVOM2 measurements were performed manually by connecting it to the fluidic device. After starting the measurement, the TEER value shifted slowly, and a stable value was reached mostly within several seconds, but sometimes it did not stabilize within the available measuring time. The automated TEER measurement, on the other hand, recorded the values at a specific point in time and took less than a second, and the time between two automated measurements was less than 2 min. Furthermore, the automated procedure eliminated the human factor. Another reason for the difference in measured values might be due to the employed measuring principles. The EVOM2 uses an alternating square wave current of ±10 μA at 12.5 Hz. Our electronics are based on a potentiostat measurement, so a constant AC voltage is applied rather than a constant current, which changes depending on the impedance. The impedance of the cell layer is highly dependent on the frequency [20]. Thus, even the small deviation of 0.5 Hz and the difference between square and sine signals will contribute to a deviation in the measured value. Despite these small deviations, the continuous TEER and impedance measuring method delivers more information and can be used in other dynamic platforms as well.

A comparison of the permeation coefficients between the static test in well plates and a test in the microfluidic system without a dynamic dilution showed that both coefficients were of the same value. Therefore, it can be assumed that the validated microfluidic system does not bring any artefacts in comparison with the conventional static permeation experiments. The cells behaved similarly in both systems, and permeation took place to a similar extent. The dependency of the permeation coefficient on the TEER of the tissue used for permeation has already been investigated in an earlier study [19]. It was observed that the determined permeation coefficient for sodium fluorescein changed only slightly above a certain TEER. For an in vitro cornea model, it was found that, for a TEER beyond 400 Ω cm^2^, the permeation coefficient did not change significantly. Thus, for the permeation studies presented here in the microfluidic system, it can also be assumed that a TEER of approximately 1100 Ω cm^2^, which was measured briefly in the experiment, still indicates a high permeation barrier.

The course of the acceptor concentration over time is shown in Figure 8. The experimental setup with its fluidic device was developed to simulate an in vivo near dynamic dilution of drug preparations applied to or in the body on single cell lines or tissue models proven in the cell culture laboratory without the need for animal experiments. The dynamic design of the microfluidic devices offers a wide range of possible applications. By adjusting the flow rate, it is possible to simulate flow conditions in different tissues; for example, the permeation of active ingredients through the blood–brain barrier or the transport of drugs after application in intestinal and nasal mucosa. The dynamic dilution rate can also be changed, so permeations in which the removal of the active ingredient takes place over a longer period of time can be simulated. Protocols established for a static experiment in well plates can be used with the microfluidic device at a flow rate without great additional effort to mimic different routes and modes of application more closely to in vivo situations.

## 5. Conclusions

We developed a test setup with advanced automation for permeation studies in a microfluidic environment that allows for the application of a desired dilution profile to the donor compartment and for continuous TEER and impedance recording. The introduced two-pump method allows one to freely program the concentration profile between the active component and a buffer solution, making it able to mimic different organotypic drug delivering processes. The presented setup offers the possibility of parallelization to carry out absorption studies with a larger number of samples. The ability to monitor flow rates and TEER values in real time allows for immediate feedback to the user. For reproducible testing with quality controls, the automation and the recording of the measurement results are essential. In the future, this system will also be used for other epithelial and endothelial absorption barriers to create microfluidic and thus more organotypic test conditions. In the next step, the in vivo dilution rate used in the presented proof-of-concept study will be applied to our corneal cell model. By this, we plan to investigate the in vitro/in vivo correlation of our setup. The new test platform is already a major step towards reliable test models that could, in the future, reduce and replace animal testing.

## Data Availability

Data is contained within the article.

## Abbreviations

The following abbreviations are used in this manuscript:

CE	Current electrodes
CSV	Comma Separated Values
DA	Differential amplifier
FBS	Fetal bovine serum
FPGA	Field programmable gate array
GLP	Good laboratory practice
GUI	Graphical user interface
SQL	Structured Query Language
GPIO	General purpose input/output
KRB	Krebs–Ringer buffer
MDCK	Madin–Darby canine kidney
NaHCO_3_	Sodium hydrogen carbonate
OP	Operational amplifier
PBS	Phosphate-buffered saline
PC	Polycarbonate
PCB	Printed circuit board
PTFE	Polytetrafluoroethylene
RE	Reference electrodes
SCPI	Standard Commands for Programmable Instruments
TEER	Transepithelial electrical resistance
